# Do Children with Better Inhibitory Control Donate More? Differentiating between Early and Middle Childhood and Cool and Hot Inhibitory Control

**DOI:** 10.3389/fpsyg.2017.02182

**Published:** 2017-12-13

**Authors:** Jian Hao

**Affiliations:** Beijing Key Laboratory of Learning and Cognition, School of Psychology, Capital Normal University, Beijing, China

**Keywords:** cool inhibitory control, hot inhibitory control, donating behavior, early childhood, middle childhood

## Abstract

Inhibitory control may play an important part in prosocial behavior, such as donating behavior. However, it is not clear at what developmental stage inhibitory control becomes associated with donating behavior and which aspects of inhibitory control are related to donating behavior during development in early to middle childhood. The present study aimed to clarify these issues with two experiments. In Experiment 1, 103 3- to 5-year-old preschoolers completed cool (Stroop-like) and hot (delay of gratification) inhibitory control tasks and a donating task. The results indicated that there were no relationships between cool or hot inhibitory control and donating behavior in the whole group and each age group of the preschoolers. In Experiment 2, 140 elementary school children in Grades 2, 4, and 6 completed cool (Stroop-like) and hot (delay of gratification) inhibitory control tasks and a donating task. The results showed that inhibitory control was positively associated with donating behavior in the whole group. Cool and hot inhibitory control respectively predicted donating behavior in the second and sixth graders. Therefore, the present study reveals that donating behavior increasingly relies on specific inhibitory control, i.e., hot inhibitory control as children grow in middle childhood.

## Introduction

Prosocial behavior refers to voluntary behavior that benefits other people (Eisenberg et al., 2010). Studies indicate that prosocial behavior is important for harmonious peer relationships (Greener, 2000; Warden and Mackinnon, 2003) and positive emotions (Layous et al., 2012; Aknin et al., 2015).

Ongley et al. (2014) distinguished two types of prosocial behavior, i.e., sharing and donating behavior. The dictator game (Gummerum et al., 2010) measures sharing behavior and involves allocating one's own items to anonymous recipients in one interaction. Thus, Ongley et al. (2014) considered sharing behavior in that situation to be costly, anonymous and unreciprocated. In contrast, these authors noted that donating behavior was likewise costly, anonymous and unreciprocated but was obviously different from sharing behavior. Donating behavior involves allocating items to recipients who need the items. For example, children were asked whether they were willing to donate candies to poor children in Rubin and Schneider's (1973) study. Donating behavior thus reflects caring about disadvantaged groups and has important significance for prosocial development. Therefore, the present study focused on children's donating behavior.

More important exploration lies in identifying the key factors related to donating behavior. Previous studies mainly explain prosocial behavior in terms of moral judgments (or moral reasoning) and moral emotion attribution (Eisenberg et al., 1987, 1991; Malti et al., 2009, 2010). Moral judgments and moral emotion attribution represent specific competences in the moral domain. However, it is still not clear whether prosocial behavior also requires general cognitive abilities. Executive function may be a general cognitive ability that is associated with donating behavior. Executive function refers to the control of thoughts, emotions and responses in goal-directed problem solving (Miyake et al., 2000; Hongwanishkul et al., 2005). In donating situations, the prosocial goals are to benefit disadvantaged people. However, children also have their own needs, thoughts and feelings that may conflict with the prosocial goals. Thus, executive function may be required to successfully engage in donating behavior. Close relationships between donating behavior and executive function as a general cognitive ability would further stimulate investigation into the role of other general cognitive abilities in prosocial behavior.

Executive function comprises various sub-functions (Hongwanishkul et al., 2005). Inhibitory control, one such sub-function, may be especially associated with prosocial behavior. Inhibitory control refers to the ability to suppress prepotent and interfering responses in order to achieve a goal (Carlson et al., 1998; Rothbart, 2011). Theoretically, it has been proposed that self-regulation or self-control capacities play an important role in prosocial behavior (Eisenberg, 2010; Gailliot, 2010). To achieve a prosocial goal, selfishness and impulsivity that block prosocial tendencies must be overridden (Eisenberg, 2010; Gailliot, 2010). Thus, individuals with better inhibitory control are likely to display more prosocial behavior such as donating behavior. Empirically, some studies have explored the role of inhibitory control in sharing behavior. Aguilar-Pardo et al. (2013) found that 4- to 6-year old children's inhibitory control was positively associated with their sharing behavior. Paulus et al. (2015) further reported a longitudinal association between preschoolers' inhibitory control and their sharing behavior. However, the recipients of the sharing behavior were the preschoolers' friends and disliked peers. Thus, it was not clear whether the same results would occur when the recipients were strangers. In addition, Ciairano et al. (2007) found a positive relationship between inhibitory control and cooperative behavior among elementary school children with mean ages of 7, 9, and 11 years. Despite this supporting evidence, some studies fail to reveal the relationships between inhibitory control and sharing behavior in preschool and elementary school children (Liu et al., 2016) and in 3- to 8-year old children (Smith et al., 2013). Still, the relationships between inhibitory control and other types of prosocial behavior, such as donating, need to be further explored. Moreover, the inconsistent results of previous studies may be related to two important factors.

One important factor is developmental stages. Some previous studies focus on either a mixed sample of preschoolers and school-aged children or a single sample of preschoolers or school-aged children with a wide age range. This sampling method has two shortcomings. First, it is not useful for indicating and comparing the relationships between inhibitory control and prosocial behavior in general developmental stages, namely early and middle childhood. Second, within early or middle childhood, specific relationships between inhibitory control and prosocial behavior at specific ages are difficult to reveal with this sampling method. The relationships between inhibitory control and donating behavior in early childhood may be different from those in middle childhood. According to Piaget's cognitive development theories (Piaget, 1929; Piaget and Cook, 1954), there are substantial differences in cognitive development between early and middle childhood. In early childhood, preschoolers experience centration, which may underlie their egocentrism (Rubin and Schneider, 1973). Thus, preschoolers are not good at understanding and caring about others. Although some follow-up studies indicate that preschoolers can understand others' basic mental states (Wellman et al., 2001) but that does not mean others' needs are their main concerns. In donating situations, although others' needs are apparent, they are not comparable to the preschoolers' own needs. Consequently, the preschoolers cannot spontaneously use their inhibitory control to suppress their own needs. Inhibitory control may not be associated with donating behavior in early childhood. However, starting in middle childhood, especially the age of 7 years, elementary school children begin to decenter and become less egocentric (Piaget, 1929; Piaget and Cook, 1954). In donating situations, although their own needs are still dominant, others' needs can also catch their attention and become a concern. Therefore, they can spontaneously use their inhibitory control to suppress their own needs. Children with better inhibitory control are likely to donate more. In other words, inhibitory control may play an important role in middle childhood.

A second important factor involves aspects of inhibitory control. Previous studies mainly examine the relationships between the cool aspect of inhibitory control and prosocial behavior. However, the hot aspect of inhibitory control may also be important for prosocial behavior, such as donating behavior. The distinction between cool and hot inhibitory control is based on the distinction between cool and hot executive function. Cool executive function involves abstract and decontextualized problem solving, whereas hot executive function emphasizes affective and motivational problem solving (Zelazo and Müller, 2002). Classic cool executive function tasks that assess inhibitory control are Stroop-like tasks (Groppe and Elsner, 2014; Bellagamba et al., 2015). For example, the day-night Stroop task asks children to say “day” when they see a moon card (Gerstadt et al., 1994). Classic hot executive function tasks that measure inhibitory control are delay of gratification tasks (Hongwanishkul et al., 2005; Groppe and Elsner, 2014). In the gift delay task, children are required not to touch a wrapped gift and to wait as long as possible within a given length of time (Kochanska et al., 2000). Thus, cool and hot inhibitory control are different aspects of inhibitory control. Hot inhibitory control especially reflects emotional or motivational inhibition abilities. It is still not clear whether specific aspects of inhibitory control are associated with donating behavior at specific developmental stages.

In sum, the present study aimed to examine the relationships between inhibitory control and donating behavior during childhood. Specifically, it aimed to clarify at what developmental stage inhibitory control becomes associated with donating behavior and which aspects of inhibitory control are associated with donating behavior from early to middle childhood. Two experiments were conducted to clarify these questions. Experiment 1 examined the relationships between inhibitory control and donating behavior in early childhood. Three-, four-, and five-year-old preschoolers were asked to complete a Stroop-like task and a delay of gratification task. A donating situation was then presented to them. Because of preschoolers' egocentrism, it is hypothesized that inhibitory control is not related to donating behavior in the whole group and any age group of the preschoolers. Experiment 2 further investigated whether relationships between inhibitory control and donating behavior emerged in middle childhood. Elementary school children in Grades 2, 4, and 6 were asked to complete a Stroop-like task and a delay of gratification task. A donating situation was then presented to them. Because of the reduction in egocentrism in middle childhood, it is hypothesized that inhibitory control is associated with donating behavior for elementary school children.

## Experiment 1

Experiment 1 aimed to investigate whether inhibitory control is associated with donating behavior in early childhood. Three age groups of preschoolers (3-, 4-, and 5-year-olds) were selected. Because Stroop-like tasks and delay of gratification tasks are typical cool and hot inhibitory control tasks, they were used to assess cool and hot inhibitory control, respectively. A revised day-night Stroop task (Bellagamba et al., 2015) was used to assess cool inhibitory control because the original day-night Stroop task is difficult for young preschoolers (Gerstadt et al., 1994). A delay of gratification task (Traverso et al., 2015) was used to measure hot inhibitory control. Donating behavior was assessed with a donating task (Ongley et al., 2014) in which the preschoolers had opportunities to donate stickers. For preschoolers, sticker are their favorite objects based on previous studies (Gummerum et al., 2010; Ongley and Malti, 2014). All these tasks were appropriate for the ages of the preschoolers.

### Method

#### Participants

One hundred three preschoolers were recruited from a local kindergarten. The 3-year-old group comprised 26 preschoolers (10 males, 16 females) between 3.00 and 3.92 years old (*M* = 3.66, *SD* = 0.23). The 4-year-old group consisted of 46 preschoolers (26 males, 20 females) between 4.00 and 4.92 years old (*M* = 4.42, *SD* = 0.33). The five-year-old group comprised 31 preschoolers (13 males and 18 females) between 5.00 and 5.92 years old (*M* = 5.47, *SD* = 0.31). All the preschoolers came from middle-class families. The study was approved by the Research Ethics Board of School of Psychology of Capital Normal University. Informed written consent was obtained from the parents of all the participants.

#### Measures

##### Cool inhibitory control

Cool inhibitory control was measured with a Stroop-like task (Bellagamba et al., 2015), which is a revised version of the day-night Stroop task (Gerstadt et al., 1994). The preschoolers were asked to say “blue” when they saw a red card and to say “red” when they saw a blue card. After confirming that the preschoolers could correctly recognize the colors, the experimenter asked the preschoolers to complete two training trials. If the preschoolers made correct responses, they were praised. If they responded incorrectly, the experimenter repeated the rule and asked them to answer again. Then, the preschoolers completed eight test trials in a fixed random order. Cool inhibitory control scores were calculated with the formula [(the number of correct responses – the number of errors)/total time] (Espy, 1997). Total time ranged from 12.34 to 40.93 s for the 3-year-olds, 9.00 to 56.30 s for the 4-year-olds, and 8.00–22.54 s for the 5-year-olds.

##### Hot inhibitory control

A delay of gratification task (Traverso et al., 2015) adapted from Kochanska et al. (1996) was used to assess hot inhibitory control. A colorful gift box was placed on a table and presented to the preschoolers. The preschoolers were asked to wait as long as they could before they opened the box. The latency (minute) was recorded and reflected hot inhibitory control.

##### Donating behavior

Donating behavior was measured with a donating task (Ongley et al., 2014) adapted from Knight et al. (1994). The preschoolers were given six stickers after they completed the previous tasks. The experimenter then explained that poor children lacked stickers and would be happy to receive them. A donation box with a photograph of poor children was then presented to the preschoolers. The experimenter told the preschoolers that they could choose to give stickers to the poor children or not, as they wished. The experimenter emphasized that the decision to give stickers and how many stickers they chose to give were totally voluntary. Thus, all the preschoolers clearly knew that they did not have to donate stickers if they did not want to. The preschoolers were told that they needed to put the stickers for the poor children into the donation box and put the stickers for themselves into their pockets. They were then left alone to complete the donation. The number of stickers the children donated served as the donating score, which ranged from 0 to 6.

#### Procedure

The preschoolers were individually tested in a quiet room. They were first asked to complete the Stroop-like task and the delay of gratification task. Half the preschoolers completed the Stroop-like task first, and the other half completed the delay of gratification task first. After completing the inhibitory control tasks, the preschoolers were given stickers for their participation. The donating task was then presented.

### Results and discussion

#### Developmental trends in inhibitory control and donating behavior

Descriptive statistics of Experiment 1 are shown in Table 1. Given the wide differences in the variances and score ranges of the different tasks, all the data were standardized before analysis. The group differences in inhibitory control and donating behavior were analyzed. One 4-year-old child and one 5-year-old child did not complete the donating task; thus, they were not included in the corresponding analyses. For cool inhibitory control, an ANOVA indicated a significant effect of age group, *F*_(2, 100)_ = 13.96, *p* < 0.001, η^2^ = 0.218. A *post hoc* Scheffe test indicated that the 5-year-olds performed significantly better on the Stroop-like task than did the 3-year-olds (*p* < 0.001) and the 4-year-olds (*p* < 0.001), and 3-year-olds and the 4-year-olds performed similarly (*p* = 0.663). For hot inhibitory control, there was also a significant effect of age group, *F*_(2, 100)_ = 3.19, *p* = 0.045, η^2^ = 0.060. A *post hoc* Scheffe test indicated that the 5-year-olds waited longer in the delay of gratification task than did the 3-year-olds (*p* = 0.048) but not the 4-year-olds (*p* = 0.276). The two younger groups had similar performances on this task (*p* = 0.501). In the donating task, the effect of age group was not significant, *F*_(2, 98)_ = 0.72, *p* = 0.490, η^2^ = 0.014. The numbers of stickers that were donated were similar for all three age groups.

**Table 1:** Descriptive statistics of Experiment 1.

	**3-year-olds**				**4-year-olds**				**5-year-olds**			
**Variables**	***M***	***SD***	**Skewness**	**Range**	***M***	***SD***	**Skewness**	**Range**	***M***	***SD***	**Skewness**	**Range**
Stroop	0.39	0.11	0.53	0.20–0.65	0.42	0.18	−0.01	−0.11–0.89	0.58	0.16	0.64	0.35–1.00
Delay of gratification	1.19	1.47	2.11	0.09–6.27	1.83	1.77	1.24	0.07–7.72	2.67	3.15	1.09	0.00–9.48
Donating behavior	2.15	1.80	0.77	0.00–6.00	2.69	1.84	0.28	0.00–6.00	2.40	1.89	0.50	0.00–6.00

Polynomial trend analyses found a significant age-related linear (contrast estimate = 0.14, *p* < 0.001) and quadratic trend (contrast estimate = 0.05, *p* = 0.043) in Stroop scores and a significant linear trend in delay of gratification scores (contrast estimate = 1.04, *p* = 0.014). There were no specific developmental trends in donating behavior scores. Different development trends in inhibitory control and donating behavior imply that they may not be associated with each other in preschoolers as a whole.

#### Relationships between inhibitory control and donating behavior

The relationships between cool and hot inhibitory control and donating behavior were then analyzed. The correlation analyses are shown in Table 2. Consistent with the trend analyses, Stroop and delay of gratification scores were not significantly correlated with donating behavior scores in the whole group and any age group of the preschoolers.

**Table 2:** Correlations between donating behavior and cool and hot inhibitory control for the whole group and each age group in Experiment 1.

	**Preschoolers**	**3-year-olds**	**4-year-olds**	**5-year-olds**
**Variables**	**Donating behavior**	**Donating behavior**	**Donating behavior**	**Donating behavior**
Age	0.03	−0.28	0.04	0.07
Gender	0.05	0.20	−0.18	0.19
Stroop	0.06	0.29	−0.05	0.14
Delay of gratification	0.04	0.07	−0.11	0.14

Experiment 1 indicated that the 5-year-olds had better cool and hot inhibitory control than the 3- or 4-year-olds. These age-related increases are consistent with previous studies (Kochanska et al., 1996; Espy, 1997; Hongwanishkul et al., 2005; Bellagamba et al., 2015). Moreover, the numbers of stickers donated were similar for all three age groups. Gummerum et al. (2010) also found a similar trend in 3- to 5-year-olds' sharing behavior. More importantly, there were no relationships between cool or hot inhibitory control and donating behavior in the whole sample and any age group. These results are consistent with Liu et al. (2016), which showed no relationships between sharing behavior and cool inhibitory control in preschoolers. One possible explanation of the findings is that preschoolers' egocentrism may cause them to pay more attention to themselves than to others. Therefore, they may not spontaneously use their inhibitory control to suppress their own interests. Inhibitory control is thus not associated with donating behavior in early childhood.

## Experiment 2

Experiment 2 aimed to further clarify whether the relationships between inhibitory control and donating behavior emerge in middle childhood and whether specific aspects of inhibitory control is related to donating behavior at specific ages. According to Piaget's cognitive development theories, children become less egocentric starting at the age of 7 years. There are significant differences in cognitive development between children over the age of 7 years and preschoolers. Thus, second graders were recruited for the experiment. Furthermore, to investigate the relationships between inhibitory control and donating behavior at different ages, fourth and sixth graders were recruited. Stroop-like and delay of gratification tasks that were appropriate for school-aged children were used to test the elementary school children's cool and hot inhibitory control. Specifically, cool inhibitory control was measured with a fruit Stroop task (Archibald and Kerns, 1999). Hot inhibitory control was assessed with a delay of gratification task (Groppe and Elsner, 2014). Donating behavior was measured with Ongley et al.'s (2014) donating task. Because our pilot study indicated that cartoon pens were attractive for elementary school children, the children were given opportunities to donate cartoon pens in the donating task.

### Method

#### Participants

One hundred and forty children in the second, fourth and sixth grades participated in the experiment. They came from a local elementary school. The second-grade group comprised 53 children (34 males, 19 females) between 7.00 and 9.17 years old (*M* = 7.70, *SD* = 0.46). The fourth-grade group consisted of 43 children (21 males, 22 females) between 9.08 and 10.17 years old (*M* = 9.66, *SD* = 0.28). The sixth-grade group included 44 children (25 males and 19 females) between 11.08 and 12.50 years old (*M* = 11.61, *SD* = 0.33). All the children came from middle-class families. The study was approved by the Research Ethics Board of School of Psychology of Capital Normal University. Informed written consent was obtained from all the participants.

#### Measures

##### Cool inhibitory control

Cool inhibitory control was measured with a fruit Stroop task (Archibald and Kerns, 1999). Four pages were presented to the children in sequence. The first page comprised 15 colored rectangles that were arranged in rows. The children needed to name the colors of these rectangles as quickly as possible within 45 s. If the children finished before the time limit, they were required to start again from the beginning. If the children made an error, they were required to correct the error and then continue. On the second, third and fourth pages, the procedures were similar, except that the children were asked to respond to fruits and vegetables. The second page consisted of fruits and vegetables in their original colors. For example, bananas were colored yellow. The children were asked to name the color of each item as quickly as they could. The third page comprised fruits and vegetables that were arranged as they were on the second page; however, the fruits and vegetables on the third page had no colors. The children were asked to name the correct color of each item as quickly as possible. The fourth page was similar to the second and third pages except that the fruits and vegetables were colored incorrectly. The children were asked to name the original color of each item as quickly as they could. The number of items completed in each page was recorded. A cool inhibitory control score was obtained using the formula [page 4 – (page 1 × page 3) / (page 1 + page 3)].

##### Hot inhibitory control

Hot inhibitory control was measured with a delay of gratification task based on Groppe and Elsner (2014). Their task was adapted from Wulfert et al. (2002). In the delay of gratification task, the children needed to choose between receiving one immediate reward or more rewards 1 week later. Two types of rewards, cartoon paper clamps and cartoon notebooks that were decorated with cartoon characters, were selected as the rewards based on our pilot study. Specifically, the children could receive one cartoon paper clamp immediately or two after 1 week. Similarly, they could choose between receiving one cartoon notebook now or three later. Only the immediate reward was presented to the children. If the children chose to receive the immediate reward, the reward was given to them at once. If the children chose to wait for more rewards, the rewards were given to them a week later. For each type of reward, whether the children chose to wait was recorded. Choosing the immediate reward was scored as 0. Choosing to delay the rewards was scored as 1. The hot inhibitory control score thus ranged from 0 to 2.

##### Donating behavior

Ongley et al.'s (2014) donating task was used to assess the children's donating behavior. The task has been proven appropriate for elementary school-aged children (Ongley et al., 2014). The children were given opportunities to donate cartoon pens that were decorated with cartoon characters to poor children. They were given six cartoon pens. In addition, the children's donating time (minute) was recorded to serve as a control variable for the possible relationships between inhibitory control and donating behavior. The time started when the experimenter left the room and ended when the children reported that they had finished donating. All the other experimental procedures were similar to those of Experiment 1.

#### Procedure

The children were individually tested in a quiet room. They completed the fruit Stroop and delay of gratification tasks first. Half of the children completed the fruit Stroop task first, and the other half completed the delay of gratification task first. The children were then given cartoon pens for participating in the previous tasks and were presented with the opportunity to donate them.

### Results and discussion

#### Developmental trends in inhibitory control and donating behavior

Descriptive statistics of Experiment 2 are shown in Table 3. All the data were standardized before analysis. The performances of each age group on the experimental tasks were compared to indicate the developmental trends in inhibitory control and donating behavior. Because of class conflicts, one second grader did not complete the fruit Stroop task, one sixth grader did not complete the delay of gratification task, and two fourth graders and two sixth graders did not complete the donating task. These students were not included in the corresponding analyses. For cool inhibitory control, an ANOVA indicated a significant effect of age group, *F*_(2, 136)_ = 20.35, *p* < 0.001, η^2^ = 0.230. A *post hoc* Bonferroni test showed that the sixth graders performed significantly better on the fruit Stroop task than did the second and fourth graders, *p* < 0.001, *p* = 0.018, respectively. The fourth graders outperformed the second graders, *p* = 0.002. For hot inhibitory control, there were no significant differences between the age groups in the delay of gratification task, *F*_(2, 136)_ = 0.29, *p* = 0.750, η^2^ = 0.004. For donating behavior, an ANOVA yielded a significant effect of age group, *F*_(2, 133)_ = 15.14, *p* < 0.001, η^2^ = 0.185. A *post hoc* Bonferroni test indicated that the sixth graders donated more than the second graders (*p* < 0.001) but not the fourth graders (*p* = 0.150). The fourth graders donated more than the second graders, *p* = 0.004. In addition, donating time did not differ between the age groups, *F*_(2, 133)_ = 0.40, *p* = 0.668, η^2^ = 0.006.

**Table 3:** Descriptive statistics of Experiment 2.

	**Second graders**				**Fourth graders**				**Sixth graders**			
**Variables**	***M***	***SD***	**Skewness**	**Range**	***M***	***SD***	**Skewness**	**Range**	***M***	***SD***	**Skewness**	**Range**
Fruit stroop	8.43	4.72	0.52	−0.50–22.51	13.08	7.16	0.31	−2.23–32.45	17.03	7.86	0.41	−0.49–36.25
Delay of gratification	1.08	0.85	−0.15	0.00–2.00	1.14	0.86	−0.28	0.00–2.00	1.00	0.85	0.00	0.00–2.00
Donating behavior	2.74	1.56	0.33	0.00–6.00	3.83	1.58	−0.11	1.00–6.00	4.52	1.67	−1.03	0.00–6.00
Donating time	0.25	0.14	1.03	0.00–0.67	0.24	0.22	2.54	0.00–1.21	0.22	0.17	1.55	0.00–0.85

Polynomial trend analyses indicated a significant age-related linear trend in fruit Stroop scores (contrast estimate = 6.08, *p* < 0.001) and no specific developmental trends in delay of gratification scores. There was also a significant linear trend in the donating behavior scores (contrast estimate = 1.26, *p* < 0.001). Similar developmental trends in cool inhibitory control and donating behavior suggest that they may be associated with each other in the elementary school children as a whole.

#### Relationships between inhibitory control and donating behavior

The correlations between cool and hot inhibitory control and donating behavior are shown in Table 4. Consistent with the trend analyses, fruit Stroop scores were positively correlated with donating behavior scores in the whole sample. The results were similar for the second graders. A scatterplot of the association is shown in the Supplementary Figure 1. However, there were no significant correlations between inhibitory control scores and donating behavior scores for the fourth graders. For the sixth graders, delay of gratification scores were positively correlated with donating behavior scores. A scatterplot of the association is shown in the Supplementary Figure 2.

**Table 4:** Correlations between donating behavior and cool and hot inhibitory control for the whole group and each age group in Experiment 2.

	**Elementary school children**	**Second graders**	**Fourth graders**	**Sixth graders**
**Variables**	**Donating behavior**	**Donating behavior**	**Donating behavior**	**Donating behavior**
Age	0.43^**^	0.01	0.35^*^	−0.07
Gender	0.01	0.18	−0.08	0.03
Donating time	−0.12	−0.31^*^	−0.06	0.03
Stroop	0.32^**^	0.32^*^	−0.01	0.14
Delay of gratification	0.17^†^	0.16	0.04	0.43^**^

† p < 0.1,

* p < 0.05,

** *p < 0.01*.

As the relationships between specific aspects of inhibitory control and donating behavior were found for the second- and sixth graders, hierarchical multiple regression analyses were conducted for the younger and older groups. The results are shown in Table 5. In each regression analysis, age, gender and donating time were entered in the first step. Fruit Stroop and delay of gratification scores were entered in the second step. The dependent variable was donating behavior. For the second graders, the regression model in the first step was marginally significant, *F*_(3, 48)_ = 2.43, *p* = 0.077. The control variables accounted for 13% of the variance in donating behavior. Donating time negatively predicted donating behavior. When inhibitory control variables were entered in the second step, the regression model was significant, *F*_(5, 46)_ = 3.11, *p* = 0.017. The inhibitory control variables explained an additional 12% of the variance in donating behavior. More importantly, only cool inhibitory control (the fruit Stroop task) was a significant positive predictor of donating behavior. For the sixth graders, the regression model in the first step failed to reach significance, *F*_(3, 37)_ = 0.74, *p* = 0.536. The control variables accounted for 6% of the variance in donating behavior. After the inhibitory control variables were entered in the second step, the regression model became significant, *F*_(5, 35)_ = 2.54, *p* = 0.046. An additional 21% of the variance in donating behavior was explained by the inhibitory control variables. However, only hot inhibitory control (delay of gratification) significantly positively predicted donating behavior.

**Table 5:** Hierarchical multiple regression analyses predicting donating behavior for the second and sixth graders in Experiment 2.

	**Second graders**			**Sixth graders**		
**Predictors**	**Δ*R*^2^**	**Δ*F***	**β**	**Δ*R*^2^**	**Δ*F***	**β**
Step 1	0.13	2.43^†^		0.06	0.74	
Age			0.02			−0.21
Gender			0.20			0.11
Donating time			−0.32^*^			0.01
Step 2	0.12	3.71^*^		0.21	5.00^*^	
Age			−0.05			−0.17
Gender			0.23^†^			0.13
Donating time			−0.29^*^			0.03
Stroop			0.35^*^			0.19
Delay of gratification			0.07			0.42^**^

† p < 0.1,

* p < 0.05,

** *p < 0.01*.

Experiment 2 demonstrated that cool inhibitory control increased during middle childhood. Archibald and Kerns (1999) found similar results using the fruit Stroop task. Meanwhile, studies using the stop-signal tasks also report a similar developmental trend in cool inhibitory control (Williams et al., 1999; Bedard et al., 2002). In contrast, there was no significant age-related increase in hot inhibitory control, which was measured using the delay of gratification task in this experiment. Previous studies demonstrate that children's performance on a typical hot executive function task, the Iowa gambling task, is also relatively stable in middle childhood (Smith et al., 2012). In addition, the results showed that children's donating behavior developed quickly during middle childhood, which is consistent with previous studies (Skarin and Moely, 1976; Froming et al., 1983). This trend may be related to increased altruistic motivation. Older children have more advanced levels of motivation for altruistic behavior, such as personal willingness to share (Bar-Tal et al., 1980).

Moreover, inhibitory control was positively associated with donating behavior in middle childhood. Ciairano et al. (2007) also found a positive relationship between cool inhibitory control and cooperative behavior in elementary school children. When children enter middle childhood, they generally shed their egocentrism. They pay attention to both their own needs and those of others. Thus, children with better inhibitory control can spontaneously suppress their own interests and make a more generous donation. Experiment 2 also showed that specific aspects of inhibitory control were related to donating behavior at specific ages. The second graders' donating behavior was predicted by their cool inhibitory control, whereas the sixth graders' donating behavior was predicted by their hot inhibitory control.

## General discussion

The present study explored whether inhibitory control was associated with donating behavior during development in early to middle childhood. Experiment 1 indicated that inhibitory control was not related to donating behavior in early childhood. Experiment 2 further indicated that inhibitory control was related to donating behavior in middle childhood. Moreover, cool inhibitory control specifically predicted donating behavior during the early stage of middle childhood, whereas hot inhibitory control specifically predicted donating behavior during the later stage of middle childhood.

Classic and age-appropriate Stroop-like and delay of gratification tasks were used to assess cool and hot inhibitory control, respectively, in the present study. The age-related trends generally replicated those of previous studies. The donating task used in the present study is also widely adopted in previous studies (e.g., Rubin and Schneider, 1973; Ongley et al., 2014). For the preschoolers and elementary school children, stickers and pens were their favorite objects respectively. They clearly knew that poor children lacked these objects and would be happy to receive them. Moreover, the experimenter clearly emphasized that whether and how many items to donate was totally voluntary. The children were left alone to complete their donation. Thus, the donating task measured the children's donating behavior, which was based on their willingness. The results found that donating behavior was limited in early childhood but increased significantly in middle childhood. Previous studies also show that from early to middle childhood, children's donating behavior develops substantially (Froming et al., 1983; Ongley et al., 2014). Thus, the development trend in donating behavior in the present study is consistent with previous findings.

The main focus of the present study was the relationships between inhibitory control and donating behavior. The results demonstrated that these relationships differed considerably between early childhood and middle childhood. Inhibitory control and donating behavior were not related to each other in early childhood but were closely connected in middle childhood. Unlike preschoolers, who are characterized by egocentrism, elementary school children are less egocentric and are able to pay more attention to others' needs. Previous studies also confirm that elementary school children produce more moral justifications during moral evaluations than preschoolers do, and their moral justifications involve the welfare of others (Malti et al., 2009; Ongley et al., 2014). In contrast, preschoolers mention more hedonistic justifications that focus on their personal needs (Malti et al., 2009). Only when others' needs are noticed can children spontaneously use their inhibitory control to suppress their own needs. Children with better inhibitory control are thus likely to display more donating behavior. Therefore, relationships between inhibitory control and donating behavior were found in middle childhood. In addition, according to Piaget's moral development stages (Piaget, 1932), preschoolers are at the heteronomous stage of moral development, whereas elementary school children are generally at the autonomous stage. Consequently, elementary school children rely on more internal self-control to carry out donating behavior.

The present study further revealed that during middle childhood, inhibitory control was positively associated with donating behavior, but specific aspects of inhibitory control were related to donating behavior at specific ages. First, there were positive relationships between inhibitory control and donating behavior in the whole group of elementary school children. Telzer et al. (2011) found that participants spent more time making decisions under a costly donation condition. They concluded that more effort may be required for costly donating. Neural evidence further demonstrates that children's prosocial behavior is positively related to the cortical thickness in their brain regions of inhibitory control (Thijssen et al., 2015). In adults, prosocial decision-making recruits brain regions related to self-control (Telzer et al., 2011). Therefore, inhibitory control is required to carry out donating behavior.

Second, cool inhibitory control predicted donating behavior in young elementary school children, whereas hot inhibitory control specifically predicted donating behavior in older elementary school children. During middle childhood, children's self-conscious emotions and emotion understanding increase with their self-awareness (Berk, 2005). Studies indicate that at this period, empathetic skills (Lonigro et al., 2014; Schwenck et al., 2014) and the abilities to recognize basic emotions and understand and experience mixed emotions (Leppänen and Hietanen, 2001; Zajdel et al., 2013) increase with age. Thus, in the donating situation, older elementary school children may not only empathize with the poor children but may also have strong emotional responses to their own gains and losses. Decision-making in a donating situation may be thus an affective process for older elementary school children. In addition, children increasingly make social comparisons when they enter middle childhood (Berk, 2005). Older elementary school children are likely to think about their own gains and losses more and thus have more emotional responses. Because emotional responses related to self-interests are still prepotent, hot inhibitory control is especially necessary to override them and induce donating behavior. Some may believe that young elementary school children also have strong emotions sometimes. However, only self-conscious emotions that characterize older elementary school children may induce spontaneous control of the emotions. Additionally, the results did not reveal relationships between inhibitory control and donating behavior in the fourth graders. Previous studies have found that, in middle childhood, children's concern for other's needs drops at ages 9–10 (Eisenberg et al., 1987). Children at this age cannot notice others' needs adequately and thus cannot spontaneously use their inhibitory control to suppress their own needs. Therefore, neither cool nor hot inhibitory control is associated with donating behavior in children of this age, i.e., the fourth graders in the present study.

Despite the new findings, the present study also has some limitations. First, the results provide an executive function account of donating behavior in middle childhood. However, it is not clear whether this executive function account can be applied to other prosocial behavior. Second, the purpose of the present study is to first clarify the developmental stage at which inhibitory control is associated with donating behavior and the specific aspects of inhibitory control associated with donating behavior at specific ages. Thus, a cross-sectional design was used. Future studies should further confirm the causal relationships between inhibitory control and donating behavior with longitudinal designs. Third, it is important to further examine whether prosocial behavior is associated with other general cognitive abilities, such as intelligence. Finally, some of the tasks provided a very narrow range of scores and small variance in the present study. This might have prevented the finding of correlations between some of the variables. In addition, the sample sizes of the age groups were relatively small and this might have influenced the statistic power. Thus, future studies should use tasks with a wide range of scores and groups of large sample sizes to further examine the relationships between inhibitory control and prosocial behavior such as donating behavior.

## Conclusion

Inhibitory control is not related to donating behavior in early childhood, but they are closely connected in middle childhood. During middle childhood, cool and hot inhibitory control, respectively, predict young and older children's donating behavior. Therefore, donating behavior increasingly relies on specific inhibitory control, i.e., hot inhibitory control as children grow in middle childhood.

## Author contributions

JH proposed the concept and designed the work, performed the acquisition of data for the work, analyzed and interpreted the data and drafted the work. JH revised the work for important intellectual content. JH finally approved the version to be published. JH agreed to be accountable for all aspects of the work in terms of ensuring that questions related to the accuracy or integrity of any part of the work are appropriately investigated and resolved.

### Conflict of interest statement

The author declares that the research was conducted in the absence of any commercial or financial relationships that could be construed as a potential conflict of interest.

## References

[B1] Aguilar-Pardo D. Martínez-Arias R. Colmenares F. (2013). The role of inhibition in young children's altruistic behaviour. Cogn. Process. 14, 301–307. 10.1007/s10339-013-0552-6 23436211

[B2] Aknin L. B. Broesch T. Hamlin J. K. Van de Vondervoort J. W. (2015). Prosocial behavior leads to happiness in a small-scale rural society. J. Exp. Psychol. Gen. 144, 788–795. 10.1037/xge0000082 26030168

[B3] Archibald S. J. Kerns K. A. (1999). Identification and description of new tests of executive functioning in children. Child Neuropsychol. 5, 115–129. 10.1076/chin.5.2.115.3167

[B4] Bar-Tal D. Raviv A. Leiser T. (1980). The development of altruistic behavior: empirical evidence. Dev. Psychol. 16, 516–524. 10.1037/0012-1649.16.5.516

[B5] Bedard A. C. Nichols S. Barbosa J. A. Schachar R. Logan G. D. Tannock R. (2002). The development of selective inhibitory control across the life span. Dev. Neuropsychol. 21, 93–111. 10.1207/S15326942DN2101_5 12058837

[B6] Bellagamba F. Addessi E. Focaroli V. Pecora G. Maggiorelli V. Pace B.. (2015). False belief understanding and “cool” inhibitory control in 3-and 4-years-old Italian children. Front. Psychol. 6:872. 10.3389/fpsyg.2015.00872 26175700 PMC4483514

[B7] Berk L. E. (2005). Infants, Children, and Adolescents. Auckland: Pearson Education New Zealand.

[B8] Carlson S. M. Moses L. J. Hix H. R. (1998). The role of inhibitory processes in young children's difficulties with deception and false belief. Child Dev. 69, 672–691. 10.1111/j.1467-8624.1998.00672.x 9680679

[B9] Ciairano S. Visu-Petra L. Settanni M. (2007). Executive inhibitory control and cooperative behavior during early school years: a follow-up study. J. Abnorm. Child Psychol. 35, 335–345. 10.1007/s10802-006-9094-z 17226093

[B10] Eisenberg N. (2010). Empathy-related responding: Links with self-regulation, moral judgment, and moral behavior, in Prosocial Motives, Emotions, and Behavior: The Better Angels of Our Nature, eds Mikulincer M. Shaver P. R. (Washington, DC: American Psychological Association), 129–148.

[B11] Eisenberg N. Eggum N. D. Edwards A. (2010). Empathy-related responding and moral development, in Emotions, Aggression, and Morality In Children: Bridging Development and Psychopathology, eds Arsenio W. F. Lemerise E. A. (Washington, DC: American Psychological Association), 115–135.

[B12] Eisenberg N. Miller P. A. Shell R. McNalley S. Shea C. (1991). Prosocial development in adolescence: a longitudinal study. Dev. Psychol. 27, 849–857. 10.1037/0012-1649.27.5.849

[B13] Eisenberg N. Shell R. Pasternack J. Lennon R. Beller R. Mathy R. M. (1987). Prosocial development in middle childhood: a longitudinal study. Dev. Psychol. 23, 712–718. 10.1037/0012-1649.23.5.712

[B14] Espy K. A. (1997). The shape school: assessing executive function in preschool children. Dev. Neuropsychol. 13, 495–499. 10.1080/87565649709540690

[B15] Froming W. J. Allen L. Underwood B. (1983). Age and generosity reconsidered: cross-sectional and longitudinal evidence. Child Dev. 54, 585–593. 10.2307/1130045

[B16] Gailliot M. T. (2010). The effortful and energy-demanding nature of prosocial behavior, in Prosocial Motives, Emotions, and Behavior: The Better Angels of Our Nature, eds Mikulincer M. Shaver P. R. (Washington, DC: American Psychological Association), 169–180.

[B17] Gerstadt C. L. Hong Y. J. Diamond A. (1994). The relationship between cognition and action: performance of children 3 1/2-7 years old on a Stroop-like day-night test. Cognition 53, 129–153. 10.1016/0010-0277(94)90068-X 7805351

[B18] Greener S. H. (2000). Peer assessment of children's prosocial behaviour. J. Moral Educ. 29, 47–60. 10.1080/030572400102925

[B19] Groppe K. Elsner B. (2014). Executive function and food approach behavior in middle childhood. Front. Psychol. 5:477. 10.3389/fpsyg.2014.00447 24904466 PMC4032895

[B20] Gummerum M. Hanoch Y. Keller M. Parsons K. Hummel A. (2010). Preschoolers' allocations in the dictator game: the role of moral emotions. J. Econ. Psychol. 31, 25–34. 10.1016/j.joep.2009.09.002

[B21] Hongwanishkul D. Happaney K. R. Lee W. S. C. Zelazo P. D. (2005). Assessment of hot and cool executive function in young children: age-related changes and individual differences. Dev. Neuropsychol. 28, 617–644. 10.1207/s15326942dn2802_4 16144430

[B22] Knight G. P. Johnson L. G. Carlo G. Eisenberg N. (1994). A multiplicative model of the dispositional antecedents of a prosocial behavior: predicting more of the people more of the time. J. Pers. Soc. Psychol. 66, 178–183. 10.1037/0022-3514.66.1.178 8126647

[B23] Kochanska G. Murray K. T. Harlan E. T. (2000). Effortful control in early childhood: continuity and change, antecedents, and implications for social development. Dev. Psychol. 36, 220–232. 10.1037/0012-1649.36.2.220 10749079

[B24] Kochanska G. Murray K. Jacques T. Y. Koenig A. L. Vandegeest K. A. (1996). Inhibitory control in young children and its role in emerging internalization. Child Dev. 67, 490–507. 10.2307/1131828 8625724

[B25] Layous K. Nelson S. K. Oberle E. Schonert-Reichl K. A. Lyubomirsky S. (2012). Kindness counts: prompting prosocial behavior in preadolescents boosts peer acceptance and well-being. PLoS ONE 7:e51380. 10.1371/journal.pone.0051380 23300546 PMC3530573

[B26] Leppänen J. M. Hietanen J. K. (2001). Emotion recognition and social adjustment in school-aged girls and boys. Scand. J. Psychol. 42, 429–435. 10.1111/1467-9450.00255 11771812

[B27] Liu B. Huang Z. Xu G. Jin Y. Chen Y. Li X.. (2016). Altruistic sharing behavior in children: role of theory of mind and inhibitory control. J. Exp. Child Psychol. 141, 222–228. 10.1016/j.jecp.2015.09.010 26452508

[B28] Lonigro A. Laghi F. Baiocco R. Baumgartner E. (2014). Mind reading skills and empathy: evidence for nice and nasty ToM behaviours in school-aged children. J. Child Fam. Stud. 23, 581–590. 10.1007/s10826-013-9722-5

[B29] Malti T. Gasser L. Buchmann M. (2009). Aggressive and prosocial children's emotion attributions and moral reasoning. Aggress. Behav. 35, 90–102. 10.1002/ab.20289 18985747

[B30] Malti T. Gasser L. Gutzwiller-Helfenfinger E. (2010). Children's interpretive understanding, moral judgments, and emotion attributions: relations to social behaviour. Br. J. Dev. Psychol. 28, 275–292. 10.1348/026151009X403838 20481388

[B31] Miyake A. Friedman N. P. Emerson M. J. Witzki A. H. Howerter A. Wagar T. D. (2000). The unity and diversity of executive functions and their contributions to complex “Frontal Lobe” tasks: a latent variable analysis. Cogn. Psychol. 41, 49–100. 10.1006/cogp.1999.0734 10945922

[B32] Ongley S. F. Malti T. (2014). The role of moral emotions in the development of children's sharing behavior. Dev. Psychol. 50, 1148–1159. 10.1037/a0035191 24294881

[B33] Ongley S. F. Nola M. Malti T. (2014). Children's giving: moral reasoning and moral emotions in the development of donation behaviors. Front. Psychol. 5:458. 10.3389/fpsyg.2014.00458 24904474 PMC4033050

[B34] Paulus M. Licata M. Kristen S. Thoermer C. Woodward A. Sodian B. (2015). Social understanding and self-regulation predict pre-schoolers' sharing with friends and disliked peers: a longitudinal study. Int. J. Behav. Dev. 39, 53–64. 10.1177/0165025414537923

[B35] Piaget J. (1929). The Child's Conception of the World. Oxford; England: Harcourt, Brace.

[B36] Piaget J. (1932). The Moral Judgment of the Child. Oxford; England: Harcourt, Brace.

[B37] Piaget J. Cook M. (1954). The Construction of Reality in the Child. New York, NY: Basic Books.

[B38] Rothbart M. K. (2011). Becoming Who We Are: Temperament and Personality in Development. New York, NY: Guilford Press.

[B39] Rubin K. H. Schneider F. W. (1973). The relationship between moral judgment, egocentrism, and altruistic behavior. Child Dev. 44, 661–665. 10.2307/1128027 4730541

[B40] Schwenck C. Göhle B. Hauf J. Warnke A. Freitag C. M. Schneider W. (2014). Cognitive and emotional empathy in typically developing children: the influence of age, gender, and intelligence. Eur. J. Dev. Psychol. 11, 63–76. 10.1080/17405629.2013.808994

[B41] Skarin K. Moely B. E. (1976). Altruistic behavior: an analysis of age and sex differences. Child Dev. 47, 1159–1165. 10.2307/1128455

[B42] Smith C. E. Blake P. R. Harris P. L. (2013). I should but I won't: why young children endorse norms of fair sharing but do not follow them. PLoS ONE, 8:e59510 10.1371/journal.pone.0059510 23527210 PMC3603928

[B43] Smith D. G. Xiao L. Bechara A. (2012). Decision making in children and adolescents: impaired Iowa gambling task performance in early adolescence. Dev. Psychol. 48, 1180–1187. 10.1037/a0026342 22081879

[B44] Telzer E. H. Masten C. L. Berkman E. T. Lieberman M. D. Fuligni A. J. (2011). Neural regions associated with self control and mentalizing are recruited during prosocial behaviors towards the family. Neuroimage 58, 242–249. 10.1016/j.neuroimage.2011.06.013 21703352 PMC3276247

[B45] Thijssen S. Wildeboer A. Muetzel R. L. Bakermans-Kranenburg M. J. Marroun H. Hofman A.. (2015). Cortical thickness and prosocial behavior in school-age children: a population-based MRI study. Soc. Neurosci. 10, 571–582. 10.1080/17470919.2015.1014063 25695908

[B46] Traverso L. Viterbori P. Usai M. C. (2015). Improving executive function in childhood: evaluation of a training intervention for 5-year-old children. Front. Psychol. 6:525. 10.3389/fpsyg.2015.00525 25983706 PMC4415324

[B47] Warden D. Mackinnon S. (2003). Prosocial children, bullies and victims: an investigation of their sociometric status, empathy and social problem-solving strategies. Br. J. Dev. Psychol. 21, 367–385. 10.1348/026151003322277757

[B48] Wellman H. M. Cross D. Watson J. (2001). Meta-analysis of theory-of-mind development: the truth about false belief. Child Dev. 72, 655–684. 10.1111/1467-8624.00304 11405571

[B49] Williams B. R. Ponesse J. S. Schachar R. J. Logan G. D. Tannock R. (1999). Development of inhibitory control across the life span. Dev. Psychol. 35, 205–213. 10.1037/0012-1649.35.1.205 9923475

[B50] Wulfert E. Block J. A. Santa Ana E. Rodriguez M. L. Colsman M. (2002). Delay of gratification: impulsive choices and problem behaviors in early and late adolescence. J. Pers. 70, 533–552. 10.1111/1467-6494.05013 12095190

[B51] Zajdel R. T. Bloom J. M. Fireman G. Larsen J. T. (2013). Children's understanding and experience of mixed emotions: the roles of age, gender, and dmpathy. J. Genet. Psychol. 174, 582–603. 10.1080/00221325.2012.732125 24303574

[B52] Zelazo P. D. Müller U. (2002). Executive function in typical and atypical development, in Blackwell Handbook of Childhood Cognitive Development, ed Goswami U. (Malden: Blackwell Publishing), 445–469.

