# Supplementary material for: Do Children with Better Inhibitory Control Donate More? Differentiating between Early and Middle Childhood and Cool and Hot Inhibitory Control
Source: Front Psychol. 2017 Dec 13;8:2182. doi: 10.3389/fpsyg.2017.02182 (PMC5733552; doi:10.3389/fpsyg.2017.02182)
Supplement: Supplementary file 1 [file Table1.DOCX]

Supplementary Material

Do children with better inhibitory control donate more? Differentiating between early and middle childhood and cool and hot inhibitory control

Jian Hao*

*** Correspondence:** Jian Hao: janeh@pku.edu.cn

# Supplementary Figures and Tables

## Supplementary Figures

**
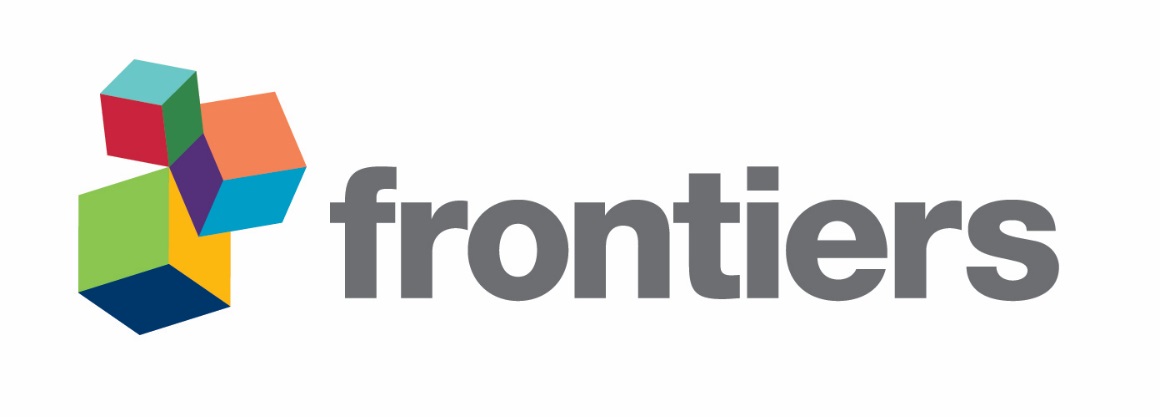
**

**Supplementary Figure 1.** The association between cool inhibitory control and donating behavior in the second graders in Experiment 2.

**Supplementary Figure 2.** The association between hot inhibitory control and donating behavior in the sixth graders in Experiment 2.
