# Supplementary figures and images for: Do Children with Better Inhibitory Control Donate More? Differentiating between Early and Middle Childhood and Cool and Hot Inhibitory Control
Source: Front Psychol. 2017 Dec 13;8:2182. doi: 10.3389/fpsyg.2017.02182 (PMC5733552; doi:10.3389/fpsyg.2017.02182)

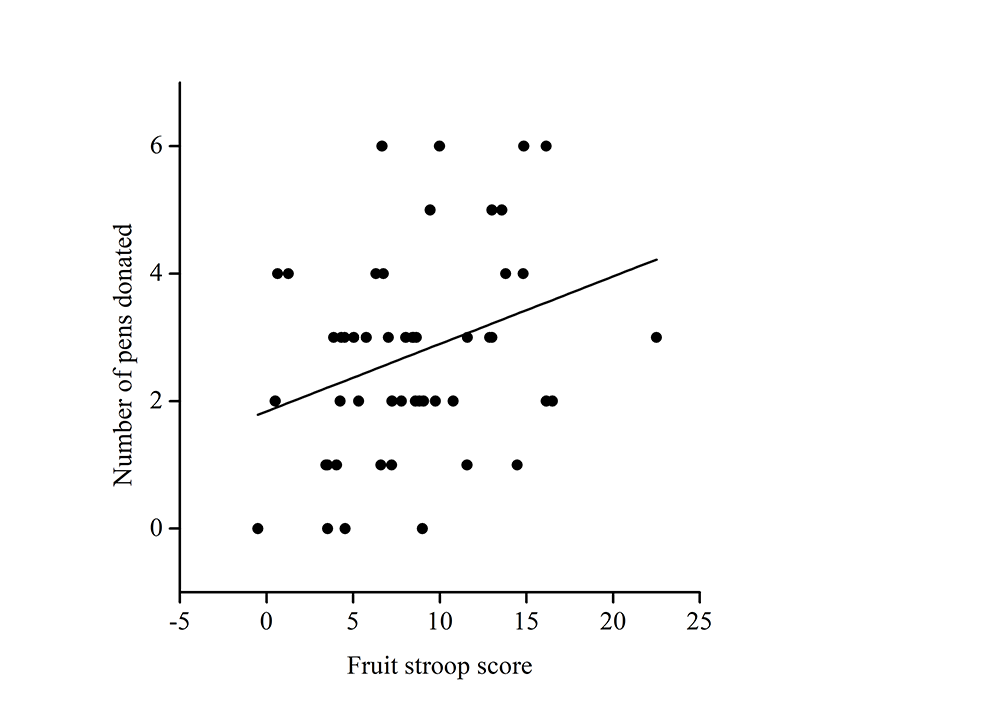

Supplement: Supplementary file 2 [file Image1.TIF]

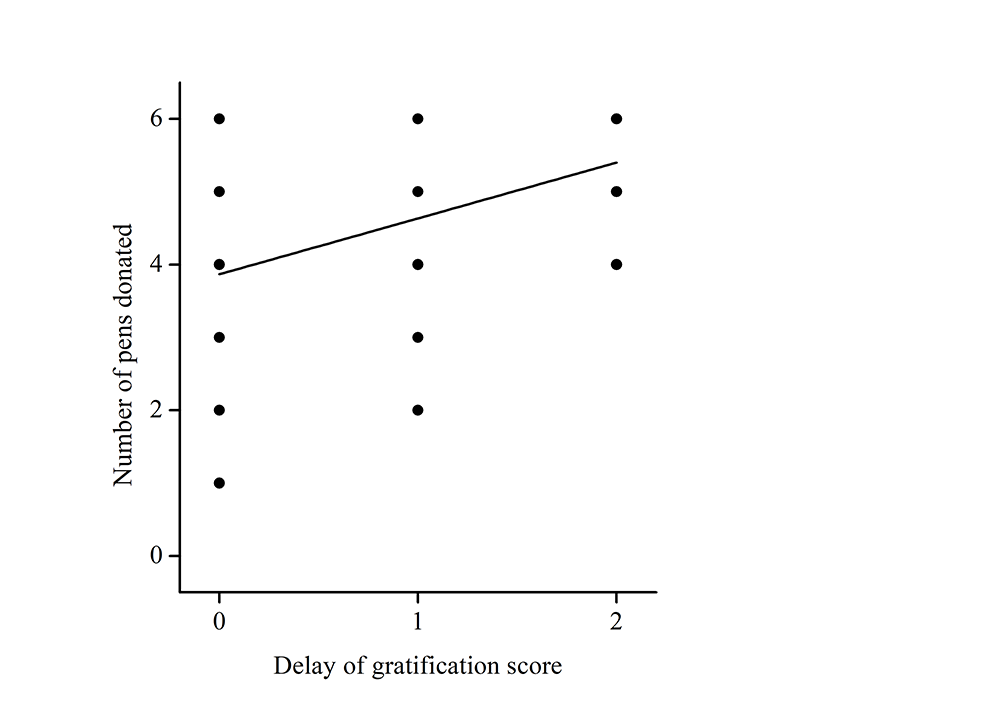

Supplement: Supplementary file 3 [file Image2.TIF]
